# Supplementary material for: Reconciling Mining with the Conservation of Cave Biodiversity: A Quantitative Baseline to Help Establish Conservation Priorities
Source: PLoS One. 2016 Dec 20;11(12):e0168348. doi: 10.1371/journal.pone.0168348 (PMC5173368; doi:10.1371/journal.pone.0168348)
Supplement: S1 Dataset — (ZIP) [file pone.0168348.s002.zip › Taxa/Serra Norte/SN_2013/N4WS/Lista N4WS-76.pdf]

CAVIDADE N4WS-0076

Classe	Ordem	Fam/Outros	Gên/Outros	Espécie	S-U	U-S	Zon.
Arachnida	Acari	(Parasitengonina)		sp.2	X		E
Arachnida	Acari	(Parasitengonina)		sp.3		X	E
Arachnida	Acari	(Parasitengonina)		sp.5	X		E
Arachnida	Acari	Laelapidae		sp.1	X		E
Arachnida	Acari	Lohmanniidae		sp.1		X	E
Arachnida	Araneae	Araneidae	*Alpaida*	*septemmammata*		X	E
Arachnida	Araneae	Ctenidae		jovem	1		E
Arachnida	Araneae	Filistatidae		jovem	X	X	E
Arachnida	Araneae	Oonopidae	aff. *Triaeris*	sp.1		X	E
Arachnida	Araneae	Pholcidae	*Leptopholcus*	sp.1		X	E/P
Arachnida	Araneae	Pholcidae	Ninetinae	sp.1		X	E
Arachnida	Araneae	Pholcidae		jovem	X		E
Arachnida	Araneae	Theridiidae		jovem	X	X	E
Arachnida	Palpigradi	Eukoeneniidae	*Eukoenenia*	sp.1	X		E
Arachnida	Pseudoscorpiones	Bochicidae		sp.1	X		E
Entognatha	Collembola	Cyphoderidae		sp.1	X		E
Entognatha	Collembola	Paronellidae		sp.1	X	X	E/P
Entognatha	Collembola	Symphypleona		sp.1	X	X	E
Entognatha	Diplura	Campodeidae		sp.1	X	X	E
Insecta	Coleoptera	Carabidae		jovem	X		E
Insecta	Coleoptera	Staphylinidae	Pselaphinae	sp.16	X		E
Insecta	Diptera	Cecidomyiidae	Cecidomyiinae	sp.		X	E/P
Insecta	Diptera	Ceratopogonidae		jovem	X		E
Insecta	Diptera	Drosophilidae		sp.	X		E
Insecta	Diptera	Ephydridae		sp.		X	E
Insecta	Diptera	Psychodidae	*Sciopemyia*	*sordellii*	X	X	E
Insecta	Heteroptera	Reduviidae	Emesinae	sp.1		2	E
Insecta	Homoptera	Cixiidae		jovem		X	E
Insecta	Hymenoptera	Formicidae	*Acromyrmex*	sp.1		X	E/P
Insecta	Hymenoptera	Formicidae	*Gnamptogenys*	*striatula*		X	E
Insecta	Hymenoptera	Formicidae	*Pachycondyla*	*striata*		3	E/P
Insecta	Hymenoptera	Formicidae	*Wasmannia*	sp.1		X	E
Insecta	Isoptera	Termitidae	*Cavitermes*	sp.		X	P
Insecta	Lepidoptera	Noctuoidea		sp.1	2	5	E
Insecta	Orthoptera	Phalangopsidae	*Paraclodes*	sp.		1	E
Insecta	Orthoptera	Phalangopsidae	*Phalangopsis*	sp.		4	P
Insecta	Psocoptera	Lepidopsocidae	*Lepidopsocus*	sp.1	X		E
Insecta	Psocoptera	Psocomorpha		jovem		X	E
Insecta	Psocoptera	Psyllipsocidae	*Psyllipsocus*	sp.3	X	X	E
Malacostraca	Isopoda	Dubioniscidae		sp.1	X		E
Malacostraca	Isopoda	Scleropactidae	*Circoniscus*	*buckupi*		X	E
Amphibia	Anura	Strabomantidae	*Pristimantis*	cf. *fenestratus*	6	3	E
Mammalia	Chiroptera	Emballonuridae	*Peropteryx*	sp.	4	7	E
